# Enzymatic Synthesis of Muconic Acid-Based Polymers: Trans, Trans-Dimethyl Muconate and Trans, β-Dimethyl Hydromuconate

**DOI:** 10.3390/polym13152498

**Published:** 2021-07-29

**Authors:** Dina Maniar, Csaba Fodor, Indra Karno Adi, Albert J. J. Woortman, Jur van Dijken, Katja Loos

**Affiliations:** 1 Macromolecular Chemistry and New Polymeric Materials, Zernike Institute for Advanced Materials, University of Groningen, Nijenborgh 4, 9747 AG Groningen, The Netherlands; d.maniar@rug.nl (D.M.); cs.fodor80@gmail.com (C.F.); indrakarno.a@gmail.com (I.K.A.); a.j.j.woortman@rug.nl (A.J.J.W.); j.van.dijken@rug.nl (J.v.D.); 2 Department of Chemistry, Analytical Chemistry Research Division, Faculty of Mathematics and Natural Sciences, Bandung Institute of Technology, Jalan Ganesha 10, Bandung 40132, Indonesia

**Keywords:** unsaturated polyester, muconic acid, enzyme catalysis, renewable resources, sustainable chemistry

## Abstract

The vast majority of commodity polymers are acquired from petrochemical feedstock, and these resources will plausibly be depleted within the next 100 years. Therefore, the utilization of carbon-neutral renewable resources for the production of polymers is crucial in modern green chemistry. Herein, we report an eco-friendly strategy that uses enzyme catalysis to design biobased unsaturated (co)polyesters from muconic acid derivatives. This method is an attractive pathway for the production of well-defined unsaturated polyesters with minimum side reactions. A suite of characterization techniques was performed to probe the reaction mechanism and properties of the obtained polyesters. It is rationalized that the alkene functionality of the muconate monomers plays an important role in the enzyme catalysis mechanism. The rendered polyesters possessed excellent thermal stabilities and unreacted alkene functionality that can consecutively undergo chain extension, copolymerization, or act as an anchor for other functional groups. These properties open new avenues in the fields of unsaturated polyester resins and photosensitive coatings.

## 1. Introduction

The use of carbon-neutral renewable resources for polymer synthesis is one of the most important endeavors in modern green chemistry [1,2,3,4,5]. Recognizing this, several research groups have directed their attention to the design of unsaturated polyesters from bio-based dicarboxylic acids, such as succinic, itaconic, fumaric, or muconic acid, with a range of various diols/polyols [6,7,8,9,10]. The alkene functionality in the polymer backbones enables these renewable-based materials to possess enhanced functionality in terms of adjustable chemical and mechanical properties, such as the glass transition temperature, biodegradability, hardness, polarity, and strength. Typically, these unsaturated polyesters are used as thermosetting resins in various industrial fields [11,12,13].

Generally, unsaturated polyesters are obtained through melt polymerization by applying conventional inorganic catalysts, such as titanium or tin alkoxides [14]. However, these methods are energy-intensive and require drastic conditions. These settings can naturally lead to undesired side reactions, including isomerization, saturation, or radical cross-linking of the alkene bonds. Alternatively, enzymatic polymerization can be used to address these issues and also other difficulties in the conventional polymerization process. Enzyme-catalyzed polymerization typically offers mild reaction conditions in terms of temperature, pressure, and pH, therefore significantly suppressing undesirable by-product formation [15,16,17]. Moreover, with enzyme catalysis, we can have perfect control of the polymer products, due to their high regio-, enantio-, chemo-, and stereoselectivity [18]. We can also expect a clean process and recyclability of the enzyme catalysts [15,16,17]. Even in many cases, the rendered polymer products have biodegradable properties as well [18]. It was also reported that this green method was successfully applied for the synthesis of other polymer classes [19,20,21,22,23,24,25,26,27,28,29,30,31]. In this overall context, polymerization reactions based on enzyme catalysis have proven to be more effective and more eco-friendly for the production of sustainable unsaturated polymers [17,32].

Among bio-based dicarboxylic acid, muconic acid (also known as 2,4-hexadienedioic acid) has received tremendous attention as a platform chemical and potential monomer precursor. *cis,cis*-Muconic acid can be derived from both sugars and lignin [33,34,35]. Normally, it can be easily converted to adipic acid (i.e., its saturated counterpart) by hydrogenation or to terephthalic acid by isomerization, followed by a reaction with ethylene and a dehydration process [34,36,37,38]. It is frequently used for commercial polymer production, such as poly(ethylene terephthalate) or nylon-6,6. The use of muconic acid as a precursor for monomers replacement is being thoroughly investigated. However, the study of its alkene functionality and its potential as functional monomer precursors is still a major challenge and deserves to be explored. The synthesis of unsaturated polymers based on muconic acid derivatives via conventional metal-catalyzed polycondensation or reversible addition–fragmentation chain transfer (RAFT) polymerization has been reported previously [8,39,40]. Recently, we reported the enzymatic polymerization of two isomers of diesters derived from muconic acid, i.e., *cis,cis* and *cis,trans*-muconate [41].

Herein, we developed an enzyme-catalyzed approach to unsaturated polyesters produced from various aliphatic diols and muconic acid derivatives, namely *trans,trans*-dimethyl muconate (*tt*MUC) and *trans,*β-dimethyl hydromuconate (TBHM). The alkene functionality in the monomer had an important effect on the reaction mechanism and enzyme catalysis. We demonstrated a controlled polymerization, which was confirmed by the unaffected alkene functionality, which enables the rendered polyesters for subsequent functionalization. The thermal properties and microstructure of the obtained unsaturated polyesters were thoroughly examined. Finally, utilizing enzymes as a catalyst for the synthesis of these unsaturated polymers, we introduced a practical route for their use as functional alternatives to petrol-derived materials with improved sustainability metrics of the materials and their production route.

## 2. Materials and Methods

Materials. *cis,cis*-Muconic acid (*cc*MA, ≥97.0%), *trans-*ß-hydromuconic acid (98%), 1,4-butanediol (4BO, 99%), 1,6-hexanediol (6DO, 99%), 1,8-octanediol (8OO, 98%), 1,10-decanediol (10DO, 99%), 1,12-dodecanediol (12DEO, 99%), dimethyl adipate (ADIP, ≥99%), dithranol (≥90%), petroleum ether (PE, bp. 30–40 °C, low-boiling-point hydrogen-treated naphtha, puriss.), molecular sieves (4Å), sodium sulfate (Na_2_SO_4_, ≥99.0%, anhydrous), sodium carbonate (Na_2_CO_3_, ≥99.5%, anhydrous), potassium trifluoroacetate (KTFA, 98%), deuterated chloroform (CDCl_3_-*d1*, 99.8 atom% D), and iodine (I_2_, ACS reagent, ≥99.8%) were purchased from Sigma-Aldrich and used without purification. Methanol (MeOH, AR grade) and ethyl acetate (EtOAc, AR grade, Biosolve) were purchased from Biosolve, and sulfuric acid (cc.H_2_SO_4_, 98%) was received from BOOM BV. Dithranol (98+%) was purchased from Fluka, and silica gel (Siliaflash P60, 40–60 μm) was ordered from Silicycle. Sodium chloride (NaCl, Emsure, for analysis) was purchased from Merck, chloroform (CHCl_3_, ChromAR, HPLC grade) was ordered from Macron, and 1,1,1,3,3,3-hexafluoro-2-propanol (HFIP, 99+%) was purchased from TCI Europe and was used without further purification. Novozyme^®^ 435 (N435, *Candida antarctica* Lipase B (CALB) immobilized on acrylic resin, 5000+ U/g) was dried under vacuum at 25 °C for 24 h before use. Diphenyl ether (DPE, 99%) was purchased from Sigma-Aldrich, distilled at 140 °C under reduced pressure, and stored with 4 Å molecular sieves under an inert atmosphere prior to use.

ATR-FTIR. Infrared spectra of the unsaturated polyesters were recorded on a Bruker VERTEX 70 spectrometer equipped with an ATR diamond single reflection plate. The measurements were performed in the range of 4000–400 cm^−1^ with 4 cm^−1^ resolution and 16 scans for each sample.

Nuclear magnetic resonance spectroscopy (NMR). NMR spectra were acquired in deuterated solvents on a Varian VXR 400 MHz (^1^H: 400 MHz; ^13^C: 100 MHz) spectrometer at room temperature. Chemical shifts (*δ*) are reported in ppm, whereas the chemical shifts are calibrated to the main solvent residual peaks. The chemical composition and the purity of the compounds were determined using CDCl3-*d1* as a solvent. The collected spectra were analyzed using MestReNova (v9.1) (Mestrelab Research S.L, Santiago, Spain).

Size exclusion chromatography (SEC). The number and weight average molecular weights (*M*_n_ and *M*_w_), as well as the dispersities (*Đ*) of the samples, were measured relative to narrow dispersity polystyrene (PSt) standards (Agilent and Polymer Laboratories) in the range from 645 to 3.0 × 10^6^ g mol^−1^ on a SEC system equipped with a Viscotek GPCmax, GPC column oven VE2585, and two PLgel MIXED-C, (5 μm × 300 mm) analytical columns from Agilent Technologies with a separation range from 200 to 2 × 10^6^ g mol^−1^ thermostatted to 35 °C in CHCl_3_ at a flow rate of 1.0 mL min^−1^ by using a Schambeck RI2012 refractive index detector. For sample preparation, the purified dry samples (10 mg) were solubilized in CHCl_3_ (3 mL) and, after, they were completely dissolved, filtered through a PTFE syringe filter (Minisart SRP 15, Sartorius Stedim Biotech, Goettingen, Germany, PTFE-membrane filter; pore size: 0.45 μm, filter diameter: 15 mm), and analyzed by SEC. The collected chromatograms were analyzed with the program OmniSEC (v5.0) (Malvern Panalytical B.V., Almelo, The Netherlands).

Matrix-assisted laser desorption/ionization-time of flight mass spectrometry (MALDI-TOF MS). Mass analysis and detection of the produced polymer microstructures were carried out on a Biosystems Voyager-DE PRO spectrometer in reflector/linear and positive mode at an acceleration voltage of 20 kV. Samples were prepared by using a premixed mixture of the dissolved matrix (dithranol, 20 mg/mL), and cationizing agent (KTFA, 5 mg/mL) dissolved in HFIP with a volume ratio of 5:1:5. The mixture (0.2–0.4 μL) was subsequently hand-spotted on the stainless-steel target and left to dry in the open air. Number average molecular weight and weight average molecular weight were calculated from $M_{n}=\sum_{i}N_{i}M_{i}/\sum_{i}N_{i}$ and $M_{w}=\sum_{i}N_{i}M_{i}^{2}/\sum_{i}N_{i}M_{i}$, respectively, where *M_i_* is the molecular weight of the chain and *N_i_* is the number of chains of that molecular weight. The mass of corresponding oligo and polyesters was calculated from $M_{p}=M_{EG}+\left(nM_{RU}\right)+M_{CI}$, where *M_P_* is the mass of the oligomer or polymer, *M_EG_* is the mass of the end groups, *n* is the number of repeating units, *M_RU_* is the mass of the repeating unit, and *M_CI_* is the mass of the counter cation. The MS data were analyzed using Data Explorer Software (v4.9) (Applied Biosystems, Waltham, MA, USA).

Differential scanning calorimetry (DSC). Glass transitions and melting points were measured on a TA-Instruments Q1000 differential scanning calorimeter under a dry nitrogen atmosphere. The samples were scanned in a temperature range from 0 °C to 120 °C by heating–cooling–heating cycles using a heating–cooling rate of 10 °C min^−1^, with isothermal sections between the cycles from 10 min. The melting temperatures (*T*_m_) were taken as the maximum of the endothermic peak, the cold crystallization temperatures (*T*_cc_) were taken as the minimum of the exothermic peak, and the melting enthalpies (*ΔH*_m_) were calculated based on the peak areas from the DSC thermograms. The resulting thermograms were evaluated with the use of Universal Analysis 2000 (v4.3A) software (TA Instruments, Hüllhorst, Germany).

Thermogravimetric analysis (TGA). Thermal stability and decomposition behavior measurements were carried out on a TA-Instruments D2500. Programmed heating from 35 °C to 750 °C was used for TG analysis at a heating rate of 10 °C min^−1^ under an inert atmosphere. The decomposition temperature (*T*_d(max)_) of the samples was assigned to the temperature of the maximum rate of weight loss. The TGA curves were analyzed by TRIOS software (v4.1) (TA Instruments).

WAXD analysis. Wide-angle X-ray diffraction (WAXD) analysis was performed at room temperature on a Bruker D8 Advance Diffractometer (Cu Kα radiation, *l* = 0.1542 nm) in the 2θ angular range of 5–50°. The accelerated voltage used was 40 kV.

POM analysis. Microscopic images of the polyesters were obtained by using a Zeiss Axiophot polarizing microscope equipped with a Sony DICC-500 camera for image acquisition. The images were recorded and processed with KS3000 software (Zeiss). The sample preparation was performed on a Mettler Toledo FP82HT hot stage and a Mettler FP90 control panel. Isothermal crystallization measurements were performed on a small fragment of the sample, inserted between two microscope cover glasses. The sample was heated to 150 °C with a heating rate of 20 °C/min, and it was then kept isothermal for 5 min at a given temperature before quenching by removing the sample from the hot stage.

### Synthetic Procedures

Synthesis of *trans,trans*-dimethyl muconates (*tt*MUC). The *cc*MA (5.0 g, 35.2 mmol) was suspended in MeOH (150 mL) with catalytic amounts of cc.H_2_SO_4_ (0.3 mL), and the mixture was refluxed for 18 h [42]. The mixture was cooled down, concentrated under vacuum, and dissolved in EtOAc. The organic phase was extracted with saturated Na_2_CO_3_ aqueous solution, followed by washing with brine. The separated organic phase was dried over Na_2_SO_4_, filtered, and concentrated (5.4 g, 31.8 mmol, 90% yield). A crude racemic mixture of dimethyl muconates (*cis,cis* and *cis,trans*) was obtained. To obtain *trans,trans-*dimethyl muconate (*tt*MUC), a isomerization reaction from the racemic mixture was performed as follows. For example, 4.50 g of the racemic mixture of dimethyl muconate, 125 mL of MeOH, and a catalytic amount of I_2_ were heated to reflux for 64 h. After that, *tt*MUC precipitated upon cooling in an ice bath. The precipitates were then filtrated, washed with cold methanol, and finally dried under vacuum. The chemical structure of the *tt*MUC was confirmed by ^1^H NMR analysis.

*Trans,trans*-dimethyl muconate (*tt*MUC): ^1^H NMR (400 MHz, chloroform-*d*): *δ* 7.32 (2H, *dd*,-COO-C***H***=CH-), 6.20 (2H, *dd*, -COO-CH=C***H***-), 3.78 (6H, s, -OC***H_3_***) ppm.

Synthesis of *trans,*β-dimethyl hydromuconate (TBHM). A catalytic amount of concentrated H_2_SO_4_ was added to a solution of *trans-*ß-hydromuconic acid (4.40 g) in MeOH (200 mL). The mixture was stirred and refluxed for 18 h, and afterward, it was concentrated using a rotary evaporator, followed by flash chromatography on silica gel with toluene as a mobile phase under steady N_2_ flow. The collected solution was then concentrated to half of the volume. Subsequently, it was washed with Na_2_CO_3_ (100 mM) and brine, and it was then dried with Na_2_SO_4(s)_. Finally, the solution was concentrated to dryness to afford TBHM as a colorless, viscous liquid. The chemical structure of the TBHM was verified by ^1^H NMR analysis.

*Trans*, β-dimethyl hydromuconate (TBHM): ^1^H NMR (400 MHz, chloroform-*d*): *δ* 5.49 (2H, *dd*, -CH_2_-C***H***=C***H***-), 2.89 (*dd*, 4H, -C***H_2_***-CH=CH-), 3.48 (s, 6H, -OC***H_3_***) ppm.

CALB-catalyzed polycondensation by temperature-varied two-stage method. The synthesis was adapted according to our method published previously [25]. Briefly, in a typical enzyme-catalyzed polymerization experiment, pre-dried N435 (15 wt% of the total monomer) and 4 Å molecular sieves (150 wt% of the total monomer) were placed in a round-bottom flask equipped with a magnetic stirring bar under an inert atmosphere. The monomers (diesters, and diols in defined ratios) and DPE solvent (500 wt% of the total monomer) were added to the catalyst under an inert atmosphere. The reaction mixture was allowed to stir slowly (200 rpm) and heated to 85 °C for 2 h under a nitrogen atmosphere and continuous low stirring speed, followed by reducing the pressure stepwise to 2 mmHg for the next 22 h. The next 24 h, the temperature was increased to 95 °C, followed by an increase to 110 °C for the last 24 h of reaction time. After the polymerization, the reaction was allowed to cool down, and the product was dissolved in CHCl_3_. N435 and molecular sieves were filtered out and the mixture was concentrated. The dissolve crude product was precipitated in excess amount of cold MeOH, separated by centrifugation, followed by decantation. The resulting polymers were dried and stored under vacuum at room temperature.

## 3. Results and Discussion

### 3.1. Monomer Synthesis

**Scheme 1:**
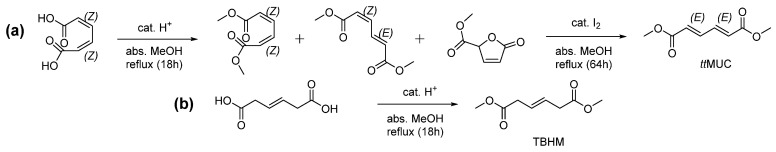
Synthesis route for the production of (**a**) *trans,trans*-dimethyl muconate (*tt*MUC) and (**b**) *trans,*β-dimethyl hydromuconate (TBHM) via esterification and isomerization.

**Figure 1:**
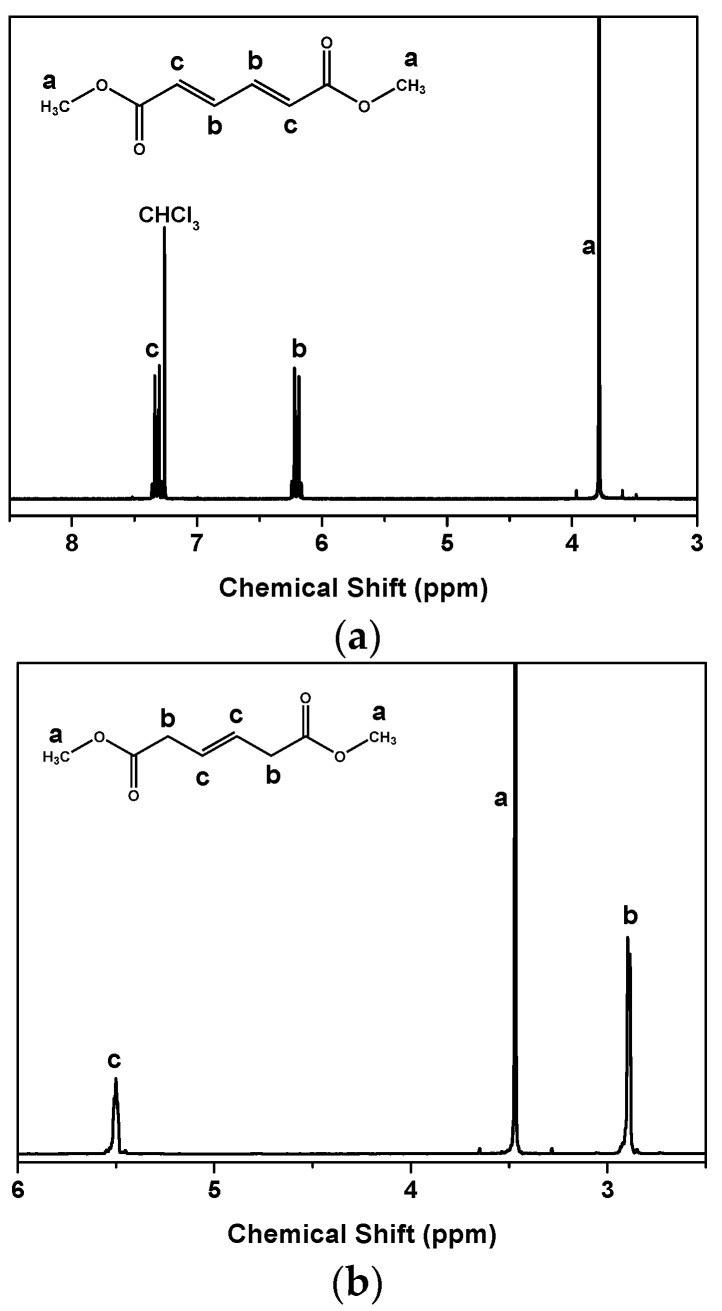
^1^H-NMR spectrum of (**a**) *trans,trans*-dimethyl muconate (*tt*MUC) and (**b**) *trans,* β-dimethyl hydromuconate (TBHM).

Unsaturated bonds in the backbone of linear polymeric materials can be desired for later modification, functionalization, and cross-linking. Hence, in this work, we studied two unsaturated muconic acid derivatives, namely *trans,trans*-dimethyl muconate (*tt*MUC) and *trans,*β-dimethyl hydromuconate (TBHM). The first monomer, *tt*MUC, was produced and isolated via the conversion of *cis, cis* muconic acid by esterification reactions to *racemic* muconic acid diester compounds, followed by I_2_-catalyzed isomerization (Scheme 1a), whereas the second monomer, TBHM, was obtained via esterification of commercial *trans,*β-hydromuconic acid (Scheme 1b). TBHM was obtained as a clear, viscous liquid. As shown in Figure 1, ^1^H-NMR analysis confirmed that the *tt*MUC and TBHM were successfully obtained. The detailed ^1^H-NMR peak assignments are available in the experimental section.

### 3.2. Enzymatic Polymerization

**Scheme 2:**
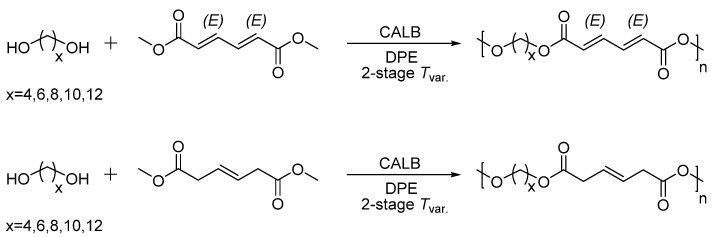
The polymerization reaction of unsaturated diesters (*tt*MUC and TBHM) with diols (with various methylene numbers between 4 and 12).

In order to determine the influence of alkene functionalities on the enzymatic polymerization, *tt*MUC and TBHM were reacted with diols of different methylene chain lengths (Scheme 2). The polycondensation reactions were conducted in total for 72 h using an immobilized *Candida antartica* Lipase B (CALB) catalyst at 85 °C under N_2_ atmosphere, followed by dynamic stepwise-reduced pressure [25]. This two-stage method was chosen because it has proved to significantly improve the polycondensation reaction, by maintaining a monophasic system and systematically removing by-products (i.e., methanol) through continuous evaporation [19].

The chemical structures of the produced unsaturated polyesters from *tt*MUC and TBHM were confirmed by ^1^H-NMR and ATR-FTIR analysis (Figures S1–S3). The detailed ^1^H-NMR peak assignments are described in Appendix A. We found that in the resulting polymers, as shown by ^1^H-NMR spectra, the alkene functionality was preserved and no stereo configurational changes were detected. This proves the stereoselectivity of the CALB enzyme and that undesired side-reactions such as isomerization, saturation, or radical cross-linking were prevented during the polymerization by utilizing the enzymatic approach.

**Figure 2:**
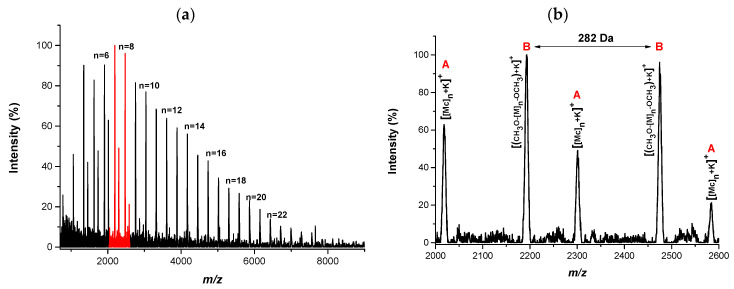

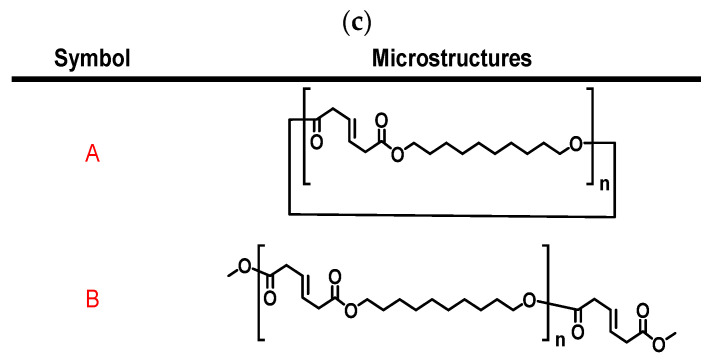
(**a**) MALDI-ToF MS spectrum of the TBHM-based polyester (entry 9 in Table 1) produced via enzymatic polymerization, (**b**) magnified part with detailed peak interpretation, and (**c**) microstructures of the obtained oligoester.

Due to the low yield obtained for the *tt*MUC-based polyester, only the microstructures and end groups of the resulting TBHM-based polyesters were analyzed by MALDI-ToF MS. By evaluating the set of peaks of the corresponding MALDI-TOF MS spectra, the TBHM polyester samples (entries 6 to 9 in Table 1) revealed three different microstructures, such as ester/ester, diol/diol, and cyclic polyester without end groups (see Figure S4). As represented in Figure 2, the end group analysis of the resulting unsaturated polyesters derived from TBHM and 1,10-decanediol (entry 9 in Table 1) revealed two different polyester species, which were ester/ester and cyclic polyesters without functional end groups. MALDI-TOF MS analysis of all TBHM samples further supports the fact that no additional side products were formed from the enzymatic polymerization.

Table 1 summarizes the molar mass and yield of the unsaturated polymers obtained by the enzymatic condensation polymerizations. The *tt*MUC diester-containing samples (Table 1, Entry 1–5) produced oligomers or low-molecular-weight polymers with number average molecular weights from 1000 to 3100 g mol^−1^ and with *Ð* of 1.22 to 1.65, while the TBHM diesters formed polymers, with molar masses up to 21,900 g mol^−1^ (Table 1, Entry 6–10). The significantly lower yields of the unsaturated polyester from *tt*MUC are attributed to their lower solubility in the applied solvent during the purification process compared to the TBHM counterparts. We note that polyesters from *tt*MUC had significantly lower molecular weights compared to their TBHM counterpart. This is due to the higher number of alkene functionality in *tt*MUC compared to TBHM. *tt*MUC cannot easily enter the active site of the CALB enzyme, possibly due to the rigidity of alkene functionality or pi stacking interactions, thus limiting further chain propagation. This is further supported by our previous report where we observed that the saturated ADIP-based polyesters possess higher number average molecular weights, while the unsaturated dimethyl muconate (*cis,cis*- and *cis,trans*-)-based polyesters could only form oligomers or low-molecular-weight polymers [41]. Furthermore, the general trend of the molecular weights increasing with the methylene numbers of the used diols is as expected. This is in agreement with the fact that CALB enzymes have a higher affinity to monomers with a longer methylene chain [43]. The result that we obtained here corroborates well with our previous work where CALB reported the highest affinity toward aliphatic monomer with eight methylene units (C_8_) [20].

**Figure 3:**
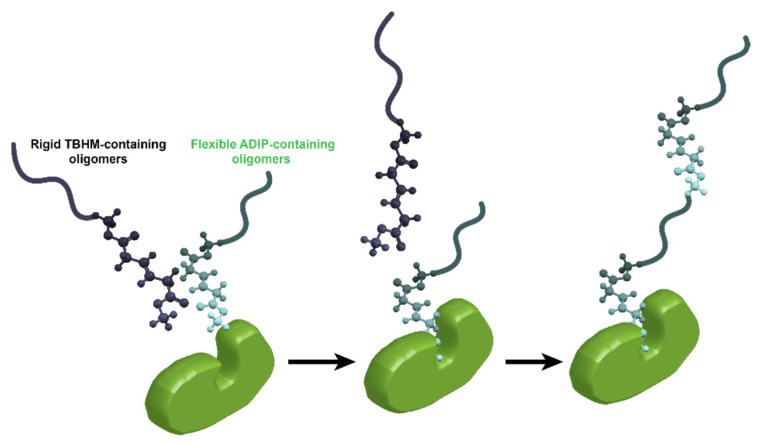
Proposed competitive propagation reaction between flexible saturated ADIP and rigid unsaturated TBHM in mixed monomer feed system. The saturated ADIP-containing oligomers are more preferable for the enzyme active site due to their flexibility.

To study if one of the diesters is preferable during the enzymatic polymerization, the competitive incorporation of saturated ADIP and unsaturated TBHM was investigated. As listed in Table 2, the polymerization of different copolymers was conducted by maintaining the same ratio of the diol (C6 and C8), with a varying ratio of the two esters (ADIP/TBHM). Slight differences in the amount of incorporated monomer compared to their feed could be observed. However, in general, the ratios of the ADIP and TBHM in the obtained copolymers were close to the expected value, similar to the feed. In general, a decreasing molar mass was observed with increasing unsaturated monomer incorporation. This can easily be observed in the case of 1,6-hexanediol where a steady decrease in molecular weight with the stable increase in TBHM incorporation (entry 14–16) was noticed. In the case of 1,8-octanediol, we see an increase in the molecular weight as we increased the TBHM feed to 37.5% (entry 13); this can be explained because the incorporated TBHM in the polymer chain was only 17.5%. From these results, one sees clear experimental evidence that, in the mixed monomer feed system where there was a competitive reaction between flexible saturated ADIP and rigid unsaturated TBHM, the saturated ADIP was more preferable and showed a higher chance to be polymerized by the enzyme (Figure 3).

### 3.3. Polymer Properties

**Figure 4:**
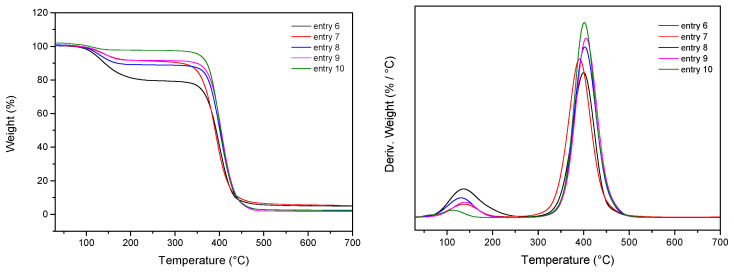
TGA and derivative thermogravimetric (DTGA) curves of TBHM-based linear polyesters (entry 6 to 10 of Table 1).

Thermogravimetric analysis (TGA) and differential scanning calorimetry (DSC) measurements were carried out to evaluate the thermal properties of the polymers. However, we only analyzed the TBHM-based polyester, due to the low yield obtained for the *tt*MUC counterpart. Figure 4 shows the TGA (DTGA) traces of the TBHM-based polyesters under nitrogen atmosphere. An initial weight loss (less than 10%) was recorded at temperatures below 200 °C due to the evaporation of the absorbed water molecules. Further weight loss was found in the temperature range between 360 and 435 °C. The DTGA traces show that the thermal degradation of the unsaturated polyesters occurred in a one-step degradation process with a maximum rate of weight loss between 390 and 400 °C, depending slightly on the methylene numbers of the used diols. The degradation process shows a near-complete decomposition profile with a low burn residue. Taken together, these results confirm that the investigated unsaturated aliphatic polyesters showed high thermal stabilities.

**Figure 5:**
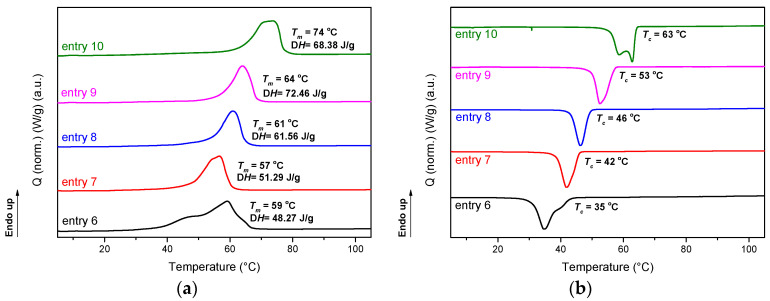
DSC thermograms: (**a**) second heating cycles and (**b**) first cooling scan of the TBHM-based polyester samples (entries 6 to 10 of Table 1).

Semi-crystalline polymers typically show both transitions corresponding to their crystalline and amorphous regions. The glass transition temperature (*T_g_*) is the property of the amorphous region where the polymers soften over a temperature range, while the crystalline region is characterized by the melting temperatures (*T_m_*) at which the ordered phase becomes a disordered phase. In general, *T_m_* values are governed by the polymer chain packing. Restriction in the polymer chain flexibility, for example, the presence of double bonds, aromatic groups, and bulky or large side groups, will usually lead to better packing, thus increasing the *T_m_* value [44]. DSC curves of TBHM-based polyesters are shown in Figure 5, where we observed the presence of *T_m_* and crystallization temperatures (*T_c_*) at around 57–74 °C and 35–63 °C, respectively. We observed that the *T_m_* and *T_c_* increased with longer methylene units in the used diol monomers. The increase in *T_m_* is due to better packing of the polyester chain with the increase in the aliphatic chain length in the repeating unit. A higher energy is needed to disrupt the chain stacking and melt the polyester, while a *T_c_* increment was observed due to the different ratios of rigid C=O units to flexible -CH_2_- units in the polyester with similar chain lengths [45]. Within the same chain length, the C=O/-CH_2_- unit ratio is important because C=O and -CH_2_- have different spatial behaviors. Consequently, a polyester with a low C=O/-CH_2_- ratio (i.e., one with a longer methylene chain) would have a higher chain uniformity and crystallize at a higher rate and temperature. However, the *T_g_* was not observed in the DSC scan of all obtained polyesters. This may be because the glass transition temperature is outside of the measured range (0–120 °C). It can also suggest that the obtained polyester is a semi-crystalline polymer material with a high degree of crystallinity.

**Figure 6:**
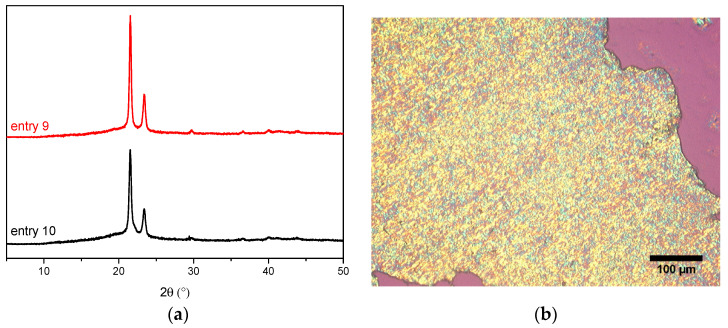
(**a**) Representative WAXD spectra of TBHM-based polymers and (**b**) POM image of polyester synthesized from TBHM and 1,12-dodecanediol (entry 10, Table 1).

To provide additional support that the obtained TBHM-based polyesters are semi-crystalline, analysis via wide-angle X-ray diffraction (WAXD) and polarized optical microscopy (POM) were conducted. Representative WAXD spectra of TBHM-based polyesters are depicted in Figure 6a. Both polyesters displayed two sharp diffraction peaks at 2θ of 21° and 23°. They possessed a similar diffraction pattern, which indicated that the polyesters had a similar crystal lattice arrangement. Polarized optical microscopy images of the polymers are shown in Figure 6b and depict a spherulitic morphology. Looking at both the WAXD and POM results confirms that the TBHM-based polyesters were indeed highly crystallized polymers.

## 4. Conclusions

In summary, we reported the enzyme-catalyzed polycondensation of different unsaturated oligo- and polyesters based on muconic acid derivatives, namely *trans,trans*-dimethyl muconate (*tt*MUC) and *trans,*β-dimethyl hydromuconate (TBHM). The enzymatic polymerization catalyzed by CALB enabled the preparation of well-defined unsaturated polyesters in a green manner with minimal side reactions, such as isomerization, saturation, or radical cross-linking. This was confirmed by ^1^H-NMR and MALDI-ToF analysis, where no peaks corresponding to side products were detected. Polyesters with lower molecular weights (i.e., up to 3100 g mol^−1^) and higher molar masses (i.e., up to 21,900 g mol^−1^) were obtained from *tt*MUC and TBHM monomers, respectively. This can be explained by the rigidity of alkene functionality or pi stacking interactions, which hamper the localization of *tt*MUC into the CALB active sites. Values of *T_m_* from 57 to 74 °C were reported, where the highest *T_m_* was possessed by the polyester synthesized from TBHM and 1,12-dodecanediol. This suggests that the TBHM-based polyesters were semi-crystalline polymers. Both WAXD and POM measurements verified the semi-crystalline properties of the obtained TBHM-based polyesters. The degradation profile showed a near-complete decomposition profile with a low burn residue and thermal stabilities up to around 400 °C.

This work not only sheds light on the synthesis mechanism of unsaturated aliphatic oligo- and polyesters via enzyme-assisted polymerization but also opens up routes for later modifications. The obtained polyesters possessed intact unreacted double bonds, which can undergo further chain extension or copolymerization with different linkers. These double bonds can also act as an anchor for small molecules or other functional groups. These properties open up potentials for their application as unsaturated polyester resins or photosensitive coatings.

## Figures and Tables

**Table 1:** Yield and molar mass of polymers obtained from the polymerization of unsaturated aliphatic diesters and diols with various carbons between 4 and 12.

Entry	Diester	Diol (C#)	Diester/Diol Ratio	Yield (%)	NMR	GPC		
					*Mn* (g mol^−1^)	*Mn* (g mol^−1^)	*Mw* (g mol^−1^)	Ð
1	ttMUC	4	50/50	5	3000	1000	1300	1.30
2	ttMUC	6	50/50	4	4600	1800	2200	1.22
3	ttMUC	8	50/50	13	4900	2700	3600	1.33
4	ttMUC	10	50/50	4	4300	2900	4800	1.65
5	ttMUC	12	50/50	2	5000	3100	5100	1.65
6	TBHM	4	50/50	49	6200	7600	16,000	2.11
7	TBHM	6	50/50	53	9900	18,600	46,500	2.50
8	TBHM	8	50/50	56	11,700	21,900	59,400	2.71
9	TBHM	10	50/50	67	5300	11,900	22,000	1.85
10	TBHM	12	50/50	76	9400	14,400	36,900	2.56

**Table 2:** Yield and molar mass of copolymers obtained from the polymerization of adipic acid, TBHM, and diols.

Entry	Diester		Diol (C#)	Feed Diester (M1/M2)/Diol Ratio	TBHM (M2) Incorporation in Polymer Chain (%)	Yield (%)	GPC		
	M1	M2					*M*_n_ (g mol^−1^)	*M*_w_ (g mol^−1^)	*Ð*
11	ADIP	TBHM	8	(37.5/12.5)/50	10.0	20	7500	12,500	1.66
12	ADIP	TBHM	8	(25/25)/50	20.5	18	6800	10,100	1.49
13	ADIP	TBHM	8	(12.5/37.5)/50	17.5	2	9100	18,800	2.07
14	ADIP	TBHM	6	(37.5/12.5)/50	9.5	40	28,000	53,600	1.92
15	ADIP	TBHM	6	(25/25)/50	20.9	9	9600	14,200	1.47
16	ADIP	TBHM	6	(12.5/37.5)/50	33.9	19	6400	8700	1.35

## Data Availability

The data presented in this study are available on request from the corresponding author.

## Supplementary Materials

The following are available online at https://www.mdpi.com/article/10.3390/polym13152498/s1, Figure S1: ^1^H NMR spectra of *tt*MUC-based linear polyesters, Figure S2: ^1^H NMR spectra of the TBHM-based linear polyesters, Figure S3: ATR-FTIR spectra of the *tt*MUC and TBHM-based linear polyesters, Figure S4: MALDI-TOF mass spectra of unsaturated polyesters derived from TBHM.

## Appendix A

Poly(butylmethylene *trans,trans* muconate): ^1^H NMR (400 MHz, chloroform-*d*): *δ* 4.23 (t, 4H, -C***H_2_***-COO-, from diol), 7.32 (m, 2H, -COO-C***H***=CH-, from *tt*MUC), 6.20 (m, 2H, -COO-CH=C***H***-, from *tt*MUC), 3.70 (s, 3H, -OC***H_3_*** from *tt*MUC end group), 1.83 (m, 4H, -COO-CH_2_-C***H_2_***-, from diol) ppm.

Poly(hexamethylene *trans,trans* muconate): ^1^H NMR (400 MHz, chloroform-*d*): *δ* 4.18 (t, 4H, -C***H_2_***-COO-, from diol), 7.30 (m, 2H, -COO-C***H***=CH-, from *tt*MUC), 6.21 (m, 2H, -COO-CH=C***H***-, from *tt*MUC), 3.70 (s, 3H, -OC***H_3_*** from *tt*MUC end group), 3.65 (t, 2H, -C***H_2_***-OH, from diol end group), 1.70 (m, 4H, -COO-CH_2_-C***H_2_***-, from diol), 1.42 (m, 4H, -COO-CH_2_-CH_2_-C***H_2_***-, from diol) ppm.

Poly(octamethylene *trans,trans* muconate): ^1^H NMR (400 MHz, chloroform-*d*): *δ* 4.17 (t, 4H, -C***H_2_***-COO-, from diol), 7.30 (m, 2H, -COO-C***H***=CH-, from *tt*MUC), 6.20 (m, 2H, -COO-CH=C***H***-, from *tt*MUC), 3.70 (s, 3H, -OC***H_3_*** from *tt*MUC end group), 3.64 (t, 2H, -C***H_2_***-OH, from diol end group), 1.69 (m, 4H, -COO-CH_2_-C***H_2_***-, from diol), 1.35 (m, 8H, -C***H_2_***-, from diol) ppm.

Poly(decamethylene *trans,trans* muconate): ^1^H NMR (400 MHz, chloroform-*d*): *δ* 4.17 (t, 4H, -C***H_2_***-COO-, from diol), 7.30 (m, 2H, -COO-C***H***=CH-, from *tt*MUC), 6.21 (m, 2H, -COO-CH=C***H***-, from *tt*MUC), 3.69 (s, 3H, -OC***H_3_*** from *tt*MUC end group), 3.64 (t, 2H, -C***H_2_***-OH, from diol end group), 1.67 (m, 4H, -COO-CH_2_-C***H_2_***-, from diol), 1.30 (m, 12H, -C***H_2_***-, from diol) ppm.

Poly(dodecamethylene *trans,trans* muconate): ^1^H NMR (400 MHz, chloroform-*d*): *δ* 4.17 (t, 4H, -C***H_2_***-COO-, from diol), 7.30 (m, 2H, -COO-C***H***=CH-, from *tt*MUC), 6.21 (m, 2H, -COO-CH=C***H***-, from *tt*MUC), 3.69 (s, 3H, -OC***H_3_*** from *tt*MUC end group), 3.64 (t, 2H, -C***H_2_***-OH, from diol end group), 1.67 (m, 4H, -COO-CH_2_-C***H_2_***-, from diol), 1.25 (m, 16H, -C***H_2_***-, from diol) ppm.

Poly(butylmethylene *trans,*ß hydromuconate) (P4HM): ^1^H NMR (400 MHz, chloroform-*d*): *δ* 4.11 (t, 4H, -C***H_2_***-COO-, from diol), 3.09 (d, 4H, -COO-C***H_2_***-, from TBHM), 5.69 (m, 2H, -C***H***=C***H***-), 3.68 (s, 3H, -OC***H_3_*** from TBHM end group), 1.70 (m, 4H, -COO-CH_2_-C***H_2_***-, from diol) ppm.

Poly(hexamethylene *trans,*ß hydromuconate) (P6HM): ^1^H NMR (400 MHz, chloroform-*d*): *δ* 4.07 (t, 4H, -C***H_2_***-COO-, from diol), 3.09 (d, 4H, -COO-C***H_2_***-, from TBHM), 5.69 (m, 2H, -C***H***=C***H***-), 3.68 (s, 3H, -OC***H_3_*** from TBHM end group), 3.64 (t, 2H, -C***H_2_***-OH from diol end group), 1.64 (m, 4H, -COO-CH_2_-C***H_2_***-, from diol), 1.37 (m, 4H, -C***H_2_***-, from diol) ppm.

Poly(octamethylene *trans,*ß hydromuconate) (P8HM): ^1^H NMR (400 MHz, chloroform-*d*): *δ* 4.07 (t, 4H, -C***H_2_***-COO-, from diol), 3.08 (d, 4H, -COO-C***H_2_***-, from TBHM), 5.69 (m, 2H, -C***H***=C***H***-), 3.68 (s, 3H, -OC***H_3_*** from TBHM end group), 3.64 (t, 2H, -C***H_2_***-OH from diol end group), 1.62 (m, 4H, -COO-CH_2_-C***H_2_***-, from diol), 1.32 (m, 8H, -C***H_2_***-, from diol) ppm.

Poly(decamethylene *trans,*ß hydromuconate) (P10HM): ^1^H NMR (400 MHz, chloroform-*d*): *δ* 4.07 (t, 4H, -C***H_2_***-COO-, from diol), 3.08 (d, 4H, -COO-C***H_2_***-, from TBHM), 5.69 (m, 2H, -C***H***=C***H****-)*, 3.68 (s, 3H, -OC***H_3_*** from TBHM end group), 3.63 (t, 2H, -C***H_2_***-OH from diol end group), 1.62 (m, 4H, -COO-CH_2_-C***H_2_***-, from diol), 1.29 (m, 12H, -C***H_2_***-, from diol) ppm.

Poly(dodecamethylene *trans,*ß hydromuconate) (P12HM): ^1^H NMR (400 MHz, chloroform-*d*): *δ* 4.07 (t, 4H, -C***H_2_***-COO-, from diol), 3.08 (d, 4H, -COO-C***H_2_***-, from TBHM), 5.69 (m, 2H, -C***H***=C***H****-)*, 3.68 (s, 3H, -OC***H_3_*** from TBHM end group), 3.63 (t, 2H, -C***H_2_***-OH from diol end group), 1.62 (m, 4H, -COO-CH_2_-C***H_2_***-, from diol), 1.27 (m, 16H, -C***H_2_***-, from diol) ppm.
