# Supplementary material for: Enzymatic Synthesis of Muconic Acid-Based Polymers: Trans, Trans-Dimethyl Muconate and Trans, β-Dimethyl Hydromuconate
Source: Polymers (Basel). 2021 Jul 29;13(15):2498. doi: 10.3390/polym13152498 (PMC8347093; doi:10.3390/polym13152498)
Supplement: Supplementary file 1 [file polymers-13-02498-s001.zip › polymers-1316599-supplementary.pdf]

SUPPORING INFORMATION

Enzymatic Synthesis of Muconic Acid-Based Polymers: Trans, Trans-Dimethyl Muconate and Trans, β-Dimethyl Hydromuconate

Dina Maniar [1], Csaba Fodor [1], Indra Karno Adi [1,2,†], Albert J. J. Woortman [1], Jur van Dijken [1] and Katja Loos [1,*]

[1] Macromolecular Chemistry and New Polymeric Materials, Zernike Institute for Advanced Materials,
University of Groningen, Nijenborgh 4, 9747 AG Groningen, The Netherlands; d.maniar@rug.nl (D.M.); cs.fodor80@gmail.com (C.F.); indrakarno.a@gmail.com (I.K.A.); a.j.j.woortman@rug.nl (A.J.J.W.); j.van.dijken@rug.nl (J.v.D.)

[2] Department of Chemistry, Analytical Chemistry Research Division, Faculty of Mathematics and Natural Sciences, Bandung Institute of Technology, Jalan Ganesha 10, Bandung 40132, Indonesia

* Correspondence: k.loos@rug.nl; Tel.: +31-50-36-368-67

† Current Address: Dexa Development Centre, Kawasan Industri Jababeka II, Jalan Industri Selatan 7 blok PP/7, Bekasi 17550, Indonesia.

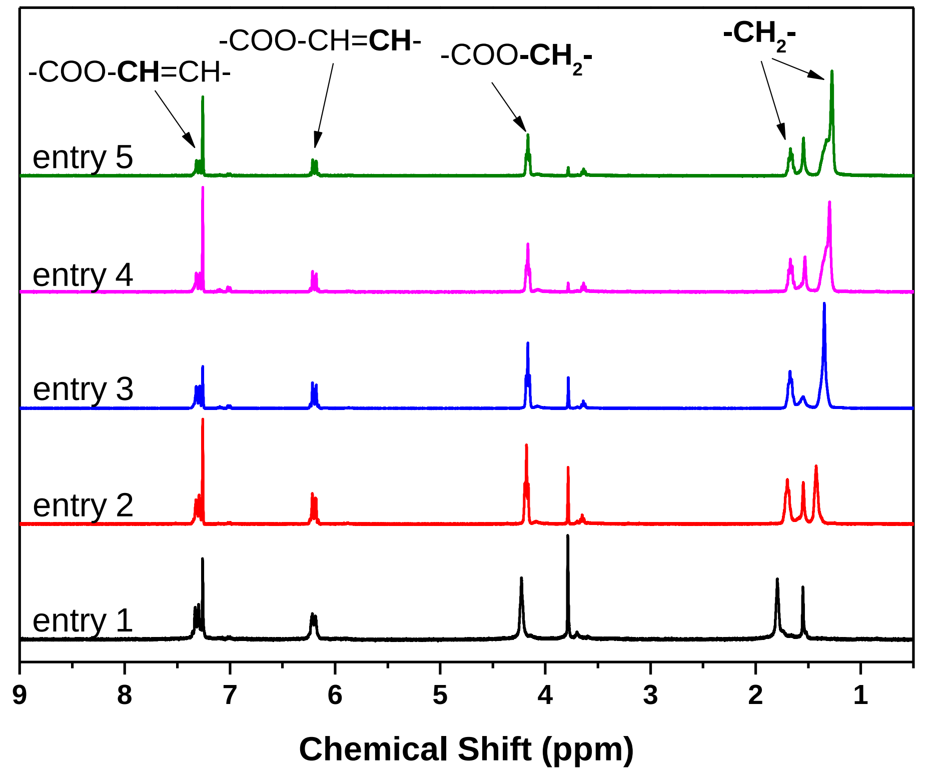

Fig. S1. ^{1}H NMR spectra of *tt*MUC based linear polyesters (chloroform-*d*, 400 MHz, RT), calibrated to to the -CH_2- peak at 4.17 ppm. Spectra are listed from the bottom to top for *tt*MUC based polyesters, entry 1 to 5.

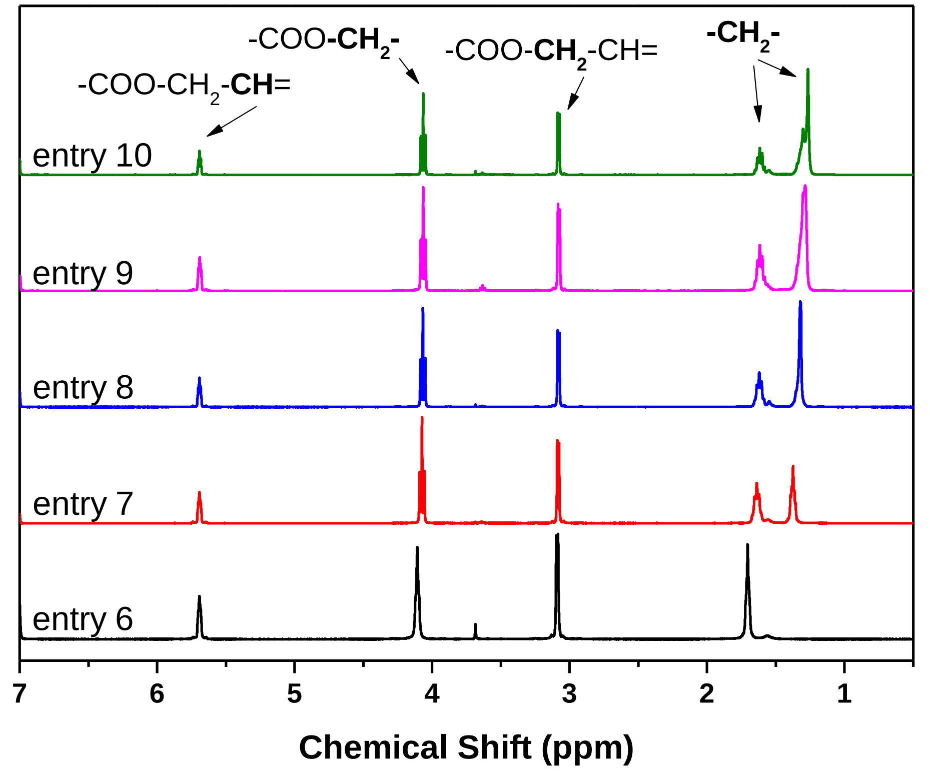

Fig. S2. ^{1}H NMR spectra of the TBHM based linear polyesters (chloroform-*d*, 400 MHz, RT), calibrated to the -CH$_2$- peak at 4.07 ppm). Spectra are listed from the bottom to top for TBHM based polyesters, entry 6 to 10.

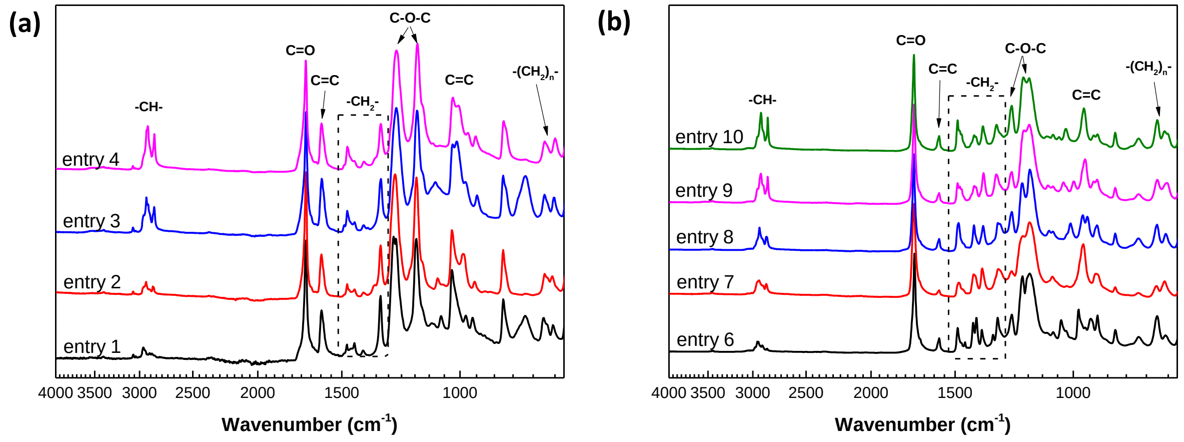

Fig. S3. ATR-FTIR spectra of the (a) *tt*MUC and (b) TBHM based linear polyesters.

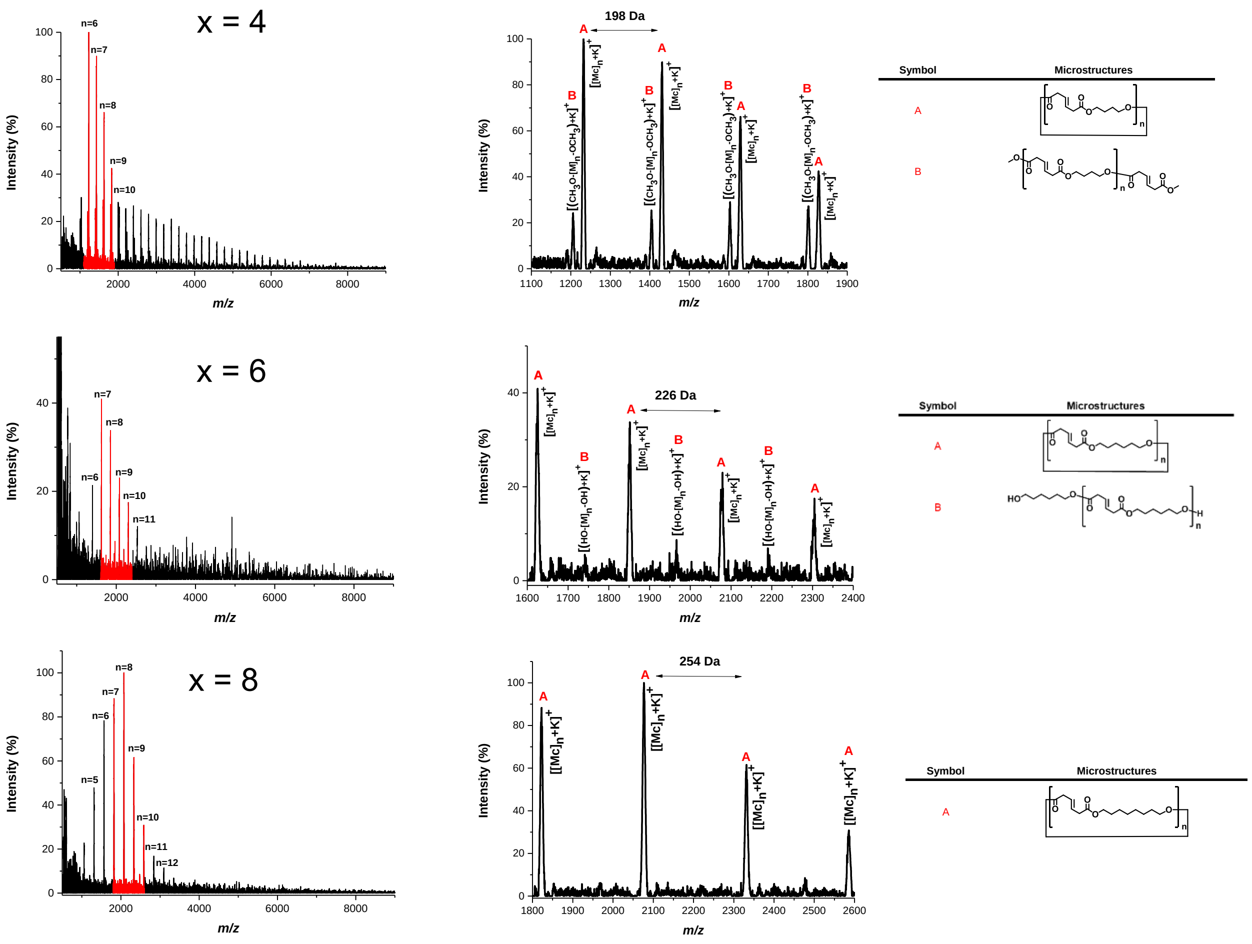

Fig. S4. MALDI-TOF mass spectra of unsaturated polyesters derived from TBHM and linear diols with various carbons between 4 and 8, the magnification of a set of peaks of the corresponding MALDI-TOF MS spectra, and the relevant microstructures of each sample (entry 6 to 8 of Table 1).
